# Abnormal Synchronization Between Cortical Delta Power and Ripples in Hippocampal Sclerosis

**DOI:** 10.1002/acn3.70032

**Published:** 2025-03-20

**Authors:** Takamitsu Iwata, Takufumi Yanagisawa, Ryohei Fukuma, Yuji Ikegaya, Satoru Oshino, Naoki Tani, Hui Ming Khoo, Hidenori Sugano, Yasushi Iimura, Hiroharu Suzuki, Haruhiko Kishima

**Affiliations:** ^1^ Department of Neurosurgery Graduate School of Medicine, Osaka University Suita Osaka Japan; ^2^ Department of Neuroinformatics, Graduate School of Medicine Osaka University Suita Osaka Japan; ^3^ Laboratory of Chemical Pharmacology, Graduate School of Pharmaceutical Sciences, The University of Tokyo Tokyo Japan; ^4^ Institute for AI and Beyond The University of Tokyo Tokyo Japan; ^5^ National Institute of Information and Communications Technology, Center for Information and Neural Networks Suita Osaka Japan; ^6^ Department of Neurosurgery Juntendo University Bunkyo‐ku Tokyo Japan

**Keywords:** delta band power, epileptogenicity, hippocampus, pathological ripple, sharp‐wave ripple

## Abstract

**Objective:**

Discriminating between epileptogenic and physiological ripples in the hippocampus is important for identifying epileptogenic (EP) zones; however, distinguishing these ripples on the basis of their waveforms is difficult. We hypothesized that the nocturnal synchronization of hippocampal ripples and cortical delta power could be used to classify epileptogenic and physiological ripples in the hippocampus.

**Methods:**

We enrolled 38 patients with electrodes implanted in the hippocampus or parahippocampal gyrus between April 2014 and March 2023 at our institution. We divided 11 patients (11 hippocampi) who were pathologically diagnosed with hippocampal sclerosis into the EP group and five patients (six hippocampi) with no epileptogenicity in the hippocampus into the nonepileptogenic (NE) group. Hippocampal ripples were detected using intracranial electroencephalography with hippocampal or parahippocampal electrodes. Cortical delta power (0.5–4 Hz) was assessed using cortical electrodes. The Pearson correlation coefficient between the ripple rates and cortical delta power (Corr‐RD) was calculated on the basis of the intracranial electroencephalographic signals recorded each night.

**Results:**

Although hippocampal ripples were similar among the EP and NE groups based on their waveforms and frequency properties, the Corr‐RDs in the EP group (mean [standard deviation]: 0.20 [0.049]) were significantly lower than those in the NE group (0.67 [0.070]). On the basis of the minimum Corr‐RDs, the two groups were classified with 94.1% accuracy.

**Interpretation:**

Our results demonstrate that the Corr‐RD is a biomarker of hippocampal epileptogenicity.

## 1 Introduction

Identifying pathological and physiological activities using intracranial electroencephalography (iEEG) is crucial for determining epileptogenic (EP) zones. High‐frequency oscillations (HFOs), which are neural activities occurring at frequencies above 80 Hz in iEEG signals, are biomarkers for epilepsy sources or pathological tissues [1, 2, 3, 4]. HFOs have been widely used to identify EP zones and predict surgical outcomes and seizure prognoses [2, 3, 4, 5, 6, 7]. HFOs are divided into two categories on the basis of frequency: ripples (80–250 Hz) and fast ripples (250–600 Hz) [1, 8]. Although fast ripples are generally considered to be more pathologically significant than ripples [7, 9], they have lower signal‐to‐noise ratios. Conversely, ripples overlap with physiological ripples, which are activated during various physiological tasks, in the high‐gamma range (80–150 Hz) [9, 10]. Therefore, discriminating between physiological and pathological HFOs is necessary to precisely evaluate the iEEG signals of patients with epilepsy; however, this discrimination is difficult [11].

In medial temporal lobe epilepsy, HFOs are linked to hippocampal sclerosis [12, 13]. More HFOs in the hippocampus are detected via intraoperative EEG in patients with hippocampal sclerosis than in patients with other epileptic conditions [12]. Additionally, ripple rates are lower in nonepileptic hippocampi than in epileptic hippocampi [13]. In the hippocampus, ripples occur due to both pathology and physiological activity. Sharp‐wave ripples (SWRs), which are ripple waves accompanied by sharp waves, are associated with cognitive functions, including memory consolidation, recall, future planning, and instructive thoughts [14, 15, 16, 17, 18, 19]. SWRs originate from synchronous neuronal firing in the CA1 region of the mammalian hippocampus [11, 15]. Inhibition of SWRs causes memory impairment, and hippocampal damage impairs recall and future planning [19, 20, 21, 22]. One hypothesis is that the crosstalk between physiological and pathological activities with similar frequency ranges may be associated with memory loss and cognitive impairment in long‐term epilepsy [12, 13]. Several studies have demonstrated that hippocampal interictal epileptic discharges disrupt memory and cognition [23, 24, 25]. The precise characterization of iEEG signals in the ripple range will contribute to identifying the pathological and physiological characteristics of the hippocampus [11].

In addition to waveform characteristics, SWRs have various physiological characteristics. Although the frequencies of SWRs fluctuate depending on the cognitive state while an individual is awake [14], they exhibit diurnal fluctuations synchronized with sleep and wake cycles, characterized by delta power in cortical activity. Notably, the SWR frequency is strongly correlated with delta power during sleep [18]. However, diurnal fluctuations in the frequencies of pathological ripples and their synchronization with delta power have not yet been elucidated. The aim of this study was to test the hypothesis that the nocturnal synchronization of hippocampal ripples and cortical delta power decreases when SWRs are contaminated by pathological ripples in the hippocampus.

## 2 Materials and Methods

### 2.1 Patients

From April 2014 to April 2023, 42 patients underwent intracranial electrode implantation in the hippocampus or parahippocampal gyrus for the focal diagnosis of refractory epilepsy. However, four patients were excluded because of disconnection of the hippocampal electrodes within 4 days of implantation (3) or lack of detailed clinical information (1) (Figure S1). The patient characteristics are summarized in Table S1. The remaining patients were divided into two groups: those with hippocampal sclerosis and those without epileptogenicity in the hippocampus. For the EP group, we selected patients who underwent focal resection after electrode implantation, had their hippocampi resected, and were pathologically diagnosed with hippocampal sclerosis. For the nonepileptogenic (NE) group, we selected patients with no significant magnetic resonance imaging (MRI) findings in the hippocampus and no seizures for at least 1 year after the resection of areas other than the hippocampus. The remaining patients were assigned to the unclassified group.

We retrospectively reviewed patient characteristics such as age, sex, side of the hippocampal electrode, electrode location, age at which the first seizure occurred, disease duration, pre‐ and postsurgical Wechsler Memory Scale‐Revised (WMSR) scores, pre‐ and postsurgical Wechsler Adult Intelligence Scale (WAIS) scores, hippocampal pathological findings, and seizure outcomes. If any index of a WAIS or WMSR component was less than 50, it was systematically adjusted to 50, given that a precise calculation of the exact value was infeasible.

### 2.2 iEEG Acquisition and Processing

The electrode placement was determined solely on the basis of clinical necessity. Our clinical team determined the best placement of the electrodes to localize the EP regions for all the patients. The EEG data analyst did not participate in this process. Data were collected from the Department of Neurosurgery at Osaka University Hospital. The research protocol was approved by the Ethical Review Board of Osaka University Hospital (approval no. 14353, UMIN000017900), and informed consent was obtained from all participants. Data were acquired from the participants during their hospital stays. The analysis included only EEG data from 4 days post‐implantation to minimize the impact of surgery on the analysis results. On the basis of visual inspection of the recorded EEG data, seizure segments were discarded from subsequent analyses. Notably, spikes unrelated to seizures were not necessarily excluded.

Intracranial EEG signals were recorded from the subdural and depth electrodes at 10 kHz using an EEG‐1200 instrument (Nihon Kohden, Tokyo, Japan). A low‐pass filter with a cutoff frequency of 3000 Hz and a high‐pass filter with a cutoff frequency of 0.016 Hz was employed for recording, and the data were downsampled to 2 kHz using an eighth‐order Chebyshev Type I low‐pass filter before resampling. For the reference electrodes, we used either subgalea electrodes facing the galea or skull‐penetrating electrodes, although intracranial electrodes were used with a few patients. The subdural contacts were arranged in both grid and strip configurations with an intercontact spacing of 10 mm and a contact diameter of 3 mm. The intercontact spacing of the deep electrode contacts ranged from 5 to 15 mm, with a width of 1 mm. Electrode localization was performed by coregistering postoperative computed tomography data with preoperative T1‐weighted or fluid‐attenuated inversion recovery MRI data using SYNAPSE VINCENT (Fujifilm, Tokyo, Japan).

### 2.3 Ripple Detection

**FIGURE 1:**
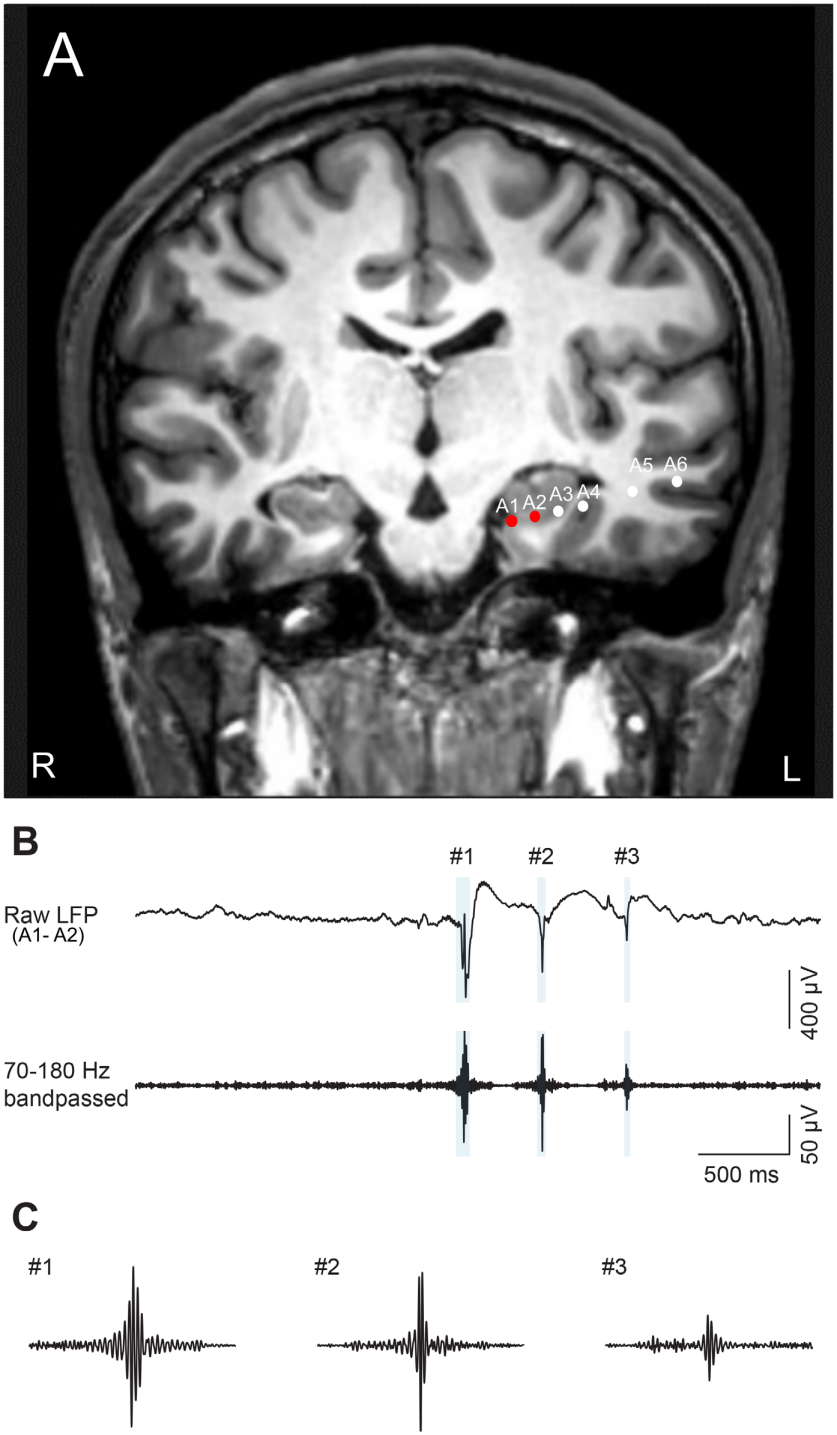
Electrodes in the hippocampus and detection of ripples. (A) An image showing preoperative magnetic resonance imaging (MRI) and postimplantation computed tomography (CT) data illustrating the electrode placement within the hippocampus. (B) Hippocampal LFPs are the bipolar potential recorded from A1–A2 (top) processed using a bandpass filter with a range of 70–180 Hz (bottom). The blue shaded area indicates the detected ripples. (C) Magnified views of three representative SWRs.

Hippocampal ripples were identified using a previously reported method [11, 18]. The local field potentials (LFPs) of the hippocampal or parahippocampal electrodes at the selected sites were converted into bipolar signals between adjacent electrodes (Figure 1A; also see Figure S2). Periods associated with epileptic activity or movement artifacts were excluded to detect the candidate ripples. To remove power‐line noise at 60, 120, 180, and 240 (±1.5) Hz, we used a 1150‐order finite impulse response (FIR) bandpass filter. The signals were subsequently filtered between 70 Hz and 180 Hz using a zero‐lag linear‐phase Hamming window FIR filter with a transition bandwidth of 5 Hz (Figure 1B,C). The instantaneous amplitude was computed using the Hilbert transformation and clipped at four times the standard deviation (SD). Finally, the clipped signal was squared and low‐pass filtered to calculate the mean and SD. The ripple candidates were selected from a range in which the unclipped signal exceeded four times the SD. Ripple candidates with durations shorter than 20 ms or longer than 200 ms were rejected, and peaks closer to 30 ms were concatenated. The detected ripple rate was normalized to the mean and SD for each day.

### 2.4 Time–Frequency Analysis of the Detected Ripples

The method outlined by Ewell et al. was applied to the dataset to assess the pathology of the detected ripples [25]. The peak slow‐wave amplitude and frequency were determined for each ripple event. To measure the slow‐wave amplitude, 500 ms data segments centered on the ripple event were bandpass filtered between 0.2 Hz and 40 Hz, and the maximum absolute amplitude of the filtered data was determined. Notably, the range used to calculate the slow‐wave amplitude is different from the cortical delta power band (0.5–4 Hz). The peak frequency was calculated by applying a fast Fourier transform to the data segments. The highest power peak at frequencies greater than 70 Hz was identified as the peak frequency. The density plots of the detected peaks for the frequency and envelope amplitudes are provided.

### 2.5 Correlation Between Hippocampal Ripples and Cortical Delta Power

To assess the correlation between hippocampal ripples and cortical delta power, delta power (0.5–4 Hz) was evaluated via electrocorticography in patients implanted with cortical electrodes (see Figure S3). For some patients implanted with stereotactic electrodes only, cortical delta power was evaluated using the LFPs from the electrodes in the area penetrating the cortex. Electrode data contaminated with artifacts were visually identified and excluded from the calculations. Bipolar potentials were obtained using all the available pairs of adjacent cortical electrodes. Periods associated with epileptic activity or movement artifacts were excluded from the analysis. The power‐line noise at 60, 120, 180, and 240 (±1.5) Hz was removed with a 1150‐order FIR band‐stop filter. To prevent edge effects, we excluded 10 s of data from either end from the analysis. These potentials were filtered using a high‐pass filter (cutoff: 0.5 Hz) and a low‐pass filter (cutoff: 4 Hz), and the Hilbert transformation was applied to determine the absolute value of the instantaneous amplitude of the analyzed signal. The ripple event rate and cortical delta power were calculated every 10 min. The delta power of each electrode pair was normalized to the mean and SD for each day and averaged. Finally, we computed the correlation coefficient between the hippocampal ripple rates and cortical delta power (Corr‐RD) every 10 min using Pearson's correlation coefficient. The calculated correlation coefficients were transformed using Fisher's z transform. These computations were performed using MATLAB 2017b.

### 2.6 Classification of Hippocampal Sclerosis Based on Corr‐RDs

To predict the pathology of the hippocampus, we classified the participants on the basis of the relationship between the hippocampal ripples and cortical delta power. We calculated each Corr‐RD value at night (from 11 pm to 7 am). Days with no measurements for > 12 h were excluded from the analysis. The Corr‐RDs varied with decreasing doses of antiepileptic drugs, and the lowest point was considered to reflect the pathological state (Table S2). We used the minimum Corr‐RD value for each participant to classify the EP and NE groups. The area under the curve (AUC) was calculated using the receiver operating characteristic (ROC) curves to assess the accuracy of the binary classification using the Corr‐RD as the threshold.

### 2.7 Statistical Analysis

Unless otherwise specified, significant differences were assessed using analysis of covariance (ANOVA), the Wilcoxon rank‐sum test, and the chi‐square test. Pearson's correlation analysis was used to evaluate correlations. The exact probability value (*p* value) was calculated as the alpha level, with *p* < 0.05 considered to indicate statistical significance. We used observational data in this study, and data collection and analysis were performed using MATLAB 2017b.

## 3 Results

### 3.1 Patient Groups

Among the 42 patients whose electrodes were implanted in the hippocampus between April 2014 and April 2023 at Osaka University Hospital, 11 patients were diagnosed with hippocampal sclerosis after hippocampal removal and were classified into the EP group (see Figure S1). The NE group included two patients in whom the hippocampus was resected without pathological abnormalities found and three patients who achieved seizure‐free status after resection of regions other than the hippocampus. The characteristics of the EP and NE groups were compared. No significant differences were observed in terms of age, sex, electrode implantation site, implantation region, electrode type, age at initial seizure, or disease duration (*p* > 0.05, Wilcoxon rank‐sum test [continuous variables] or chi‐square test [categorical variables]) (Table 1).

**TABLE 1:** Patient characteristics in the EP and NE groups.

Pt ID	Group	Age group	Sex	Epilepsy etiology	Side of electrode implantation	Implantation region	Type of electrodes	Disease duration (years)
4	EP	30	F	Left hippicampal sclerosis	Left	Parahippocampus	ECoG	23
5	EP	10	F	Right hippicampal sclerosis	Right	Hippocampus	ECoG	12
7	EP	30	F	Left hippocampal sclerosis	Left	Parahippocampus	ECoG	29
9	EP	30	M	Left hippocampal sclerosis	Left	Parahippocampus	ECoG	26
13	EP	50	M	Left hippocampal sclerosis	Left	Parahippocampus	ECoG	13
15	NE	20	M	Suspect of focal cortical dysplasia	Left	Hippocampus	ECoG	6
15	NE	20	M	Suspect of focal cortical dysplasia	Right	Hippocampus	ECoG	6
17	EP	10	F	Left hippocampal sclerosis	Left	Parahippocampus	ECoG	5
18	EP	10	M	Right hippocampal sclerosis	Right	Hippocampus	ECoG	7
20	NE	40	F	Focal cortical dysplasia (type IIb)	Right	Hippocampus	ECoG	40
22	EP	30	M	Left hippocampal sclerosis	Left	Parahippocampus	ECoG	2
23	EP	40	M	Left hippocampal sclerosis	Left	Hippocampus	ECoG	5
23	NE	40	M	Left hippocampal sclerosis	Right	Hippocampus	ECoG	5
24	NE	20	M	Polymorphous low‐grade neuroepithelial tumor of the young	Left	Parahippocampus	ECoG	8
28	EP	20	M	Left hippocampal sclerosis	Left	Parahippocampus	ECoG	14
29	NE	50	M	Unknown	Left	Hippocampus	SEEG	40
31	EP	20	F	Right hippocampal sclerosis	Right	Hippocampus	SEEG	21

### 3.2 Hippocampal Ripples of Representative Patients in the NE and EP Groups

**FIGURE 2:**
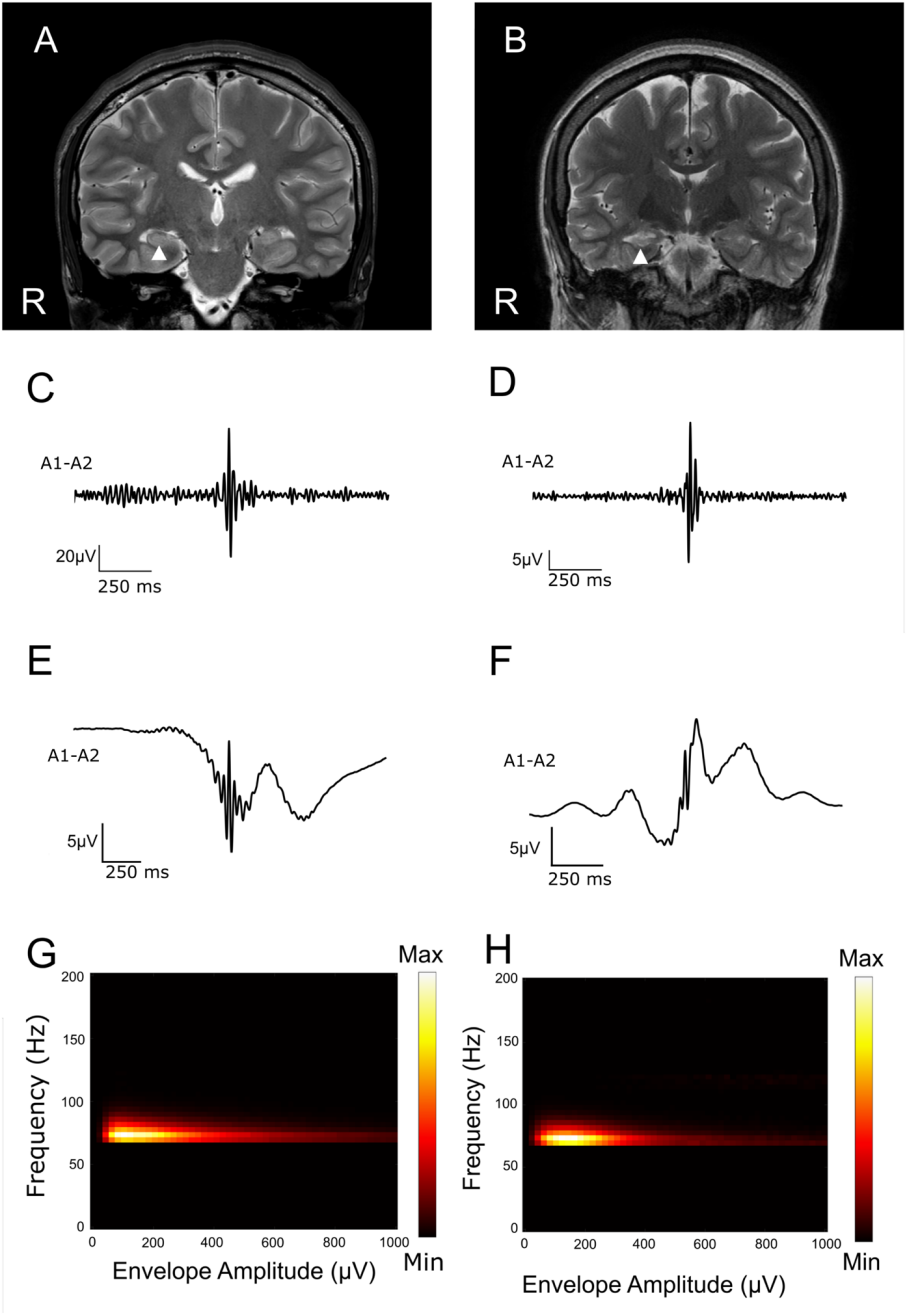
Representative hippocampal ripples detected in patients in the NE and EP groups. (A, B) Preoperative magnetic resonance imaging (MRI) data of two representative patients: Pt 15 in the NE group (A) and Pt 22 in the EP group (B). The arrowhead indicates the hippocampi, in which intracranial electrodes were implanted. The right hippocampus of Pt 22 shows hippocampal sclerosis. (C, D) Illustrative single ripple waveforms recorded with the implanted electrodes for patients in the NE group (C) and EP group (D). (E, F) Mean waveform of the field potential recorded with the implanted electrodes for patients in the NE group (E) and EP group (F). (G, H) The distributions of the peak slow‐wave amplitude and peak frequency of every ripple event are shown as a color map for the representative patients from the NE group (G) and EP group (H). The density plot reveals that all events for both patients are included in a single cluster with similar frequencies and envelope amplitudes.

Hippocampal ripples were detected in the NE and EP groups. For a representative patient in each group, the detected ripples presented features similar to those of SWRs (Figure 2A,B) [11]. The illustrative single waveforms and average waveforms of the detected ripples revealed high‐frequency ripples with sharp low‐frequency waves in both patients (Figure 2C–F). The distributions of the peak slow‐wave amplitude and peak frequency were similar between the two patients (Figure 2G,H). On the basis of the waveforms and frequency properties, the detected hippocampal ripples were similar in both patients.

### 3.3 Diurnal Fluctuations in Hippocampal Ripples

**FIGURE 3:**
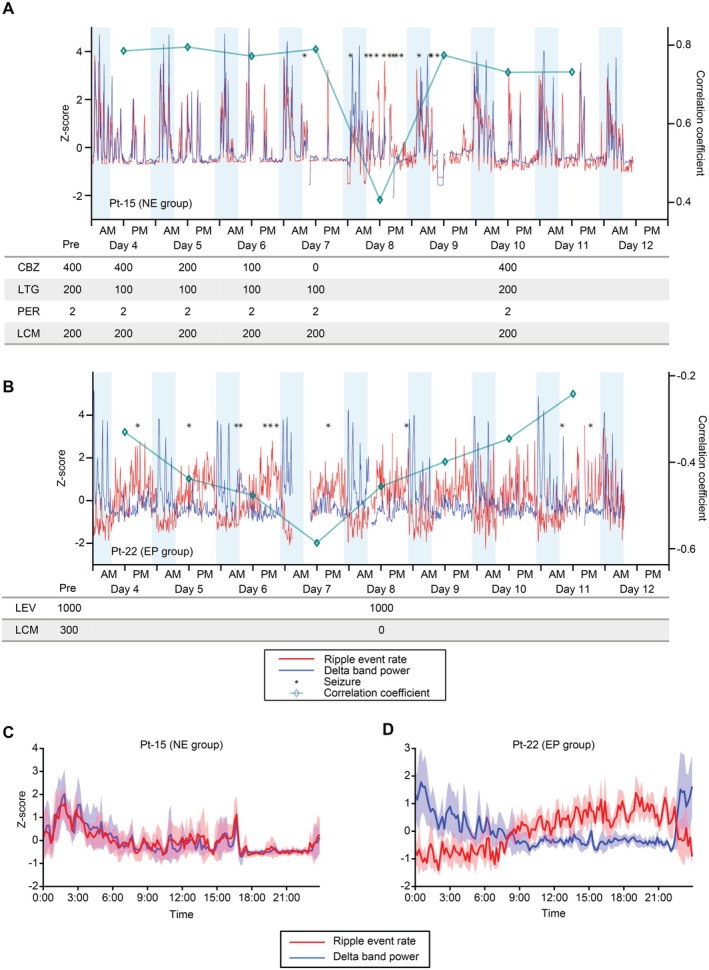
Representative examples of the hippocampal ripple event rates, cortical delta power, and seizures during 10 days of iEEG recording. (A) Time course of the hippocampal ripple event rate (red) and cortical delta power (blue) over nine consecutive days for a representative patient in the NE group (Pt 15). The light blue background indicates the time of day when the room was dark. The square symbol represents the correlation coefficient between the ripple event rate and the delta power over the whole day. The asterisk indicates the timing of the seizure. The lower column indicates the amount of antiepileptic agent provided. (B) Time course of the ripple event rate (red) and cortical delta power (blue) over nine consecutive days for a representative patient in the EP group (Pt 22). The light blue background indicates the time of day when the room was dark. The square symbol represents the correlation coefficient between the ripple event rate and the delta power over the whole day. The asterisk indicates the timing of the seizure. The lower column indicates the amount of the antiepileptic agent provided. (C, D) The lines show the mean and 95% confidence intervals of the Z‐scored ripple event rate (red) and delta power (blue) over 24 h for patients in the NE group (C) and EP group (D).

The hippocampal ripple rate and cortical delta power were assessed using long‐term iEEG recordings (Figure 3A,B). For representative patients in both the NE and EP groups, cortical delta power increased during the night and decreased during the daytime, indicating diurnal fluctuations (*p* < 0.0001, *F*_143,1034_ = 3.66, *n* = 1178 time points (NE group); *p* < 0.0001, *F*_143,997_ = 4.12, *n* = 1215 time points (EP group); one‐way ANOVA). Although antiepileptic agents were reduced during long‐term recordings, consistent diurnal fluctuations in delta power were observed throughout (Figure S4 and Table S2). Additionally, the hippocampal ripple rates of patients in the NE group were synchronized with the delta power, with high Corr‐RD values occurring each day (correlation coefficient: 0.773 [0.731–0.786]). However, the Corr‐RD value decreased on the day of the seizure. Synchronized diurnal fluctuations were observed for the average ripple rates and delta powers over the 9 days (Figure 3C and Figure S5 for the variance among channels). Conversely, for a patient in the EP group, the ripple rate increased during the daytime and decreased at night, indicating an inverse correlation with the delta power (Figure 3D, −0.419 [−0.461 to −0.342], Figure S6). Additionally, the Corr‐RD value decreased further during the withdrawal period of the antiepileptic agents in patients with seizures.

### 3.4 Corr‐RDs Differed Among Patient Groups and Changed With Seizure Frequency

**FIGURE 4:**
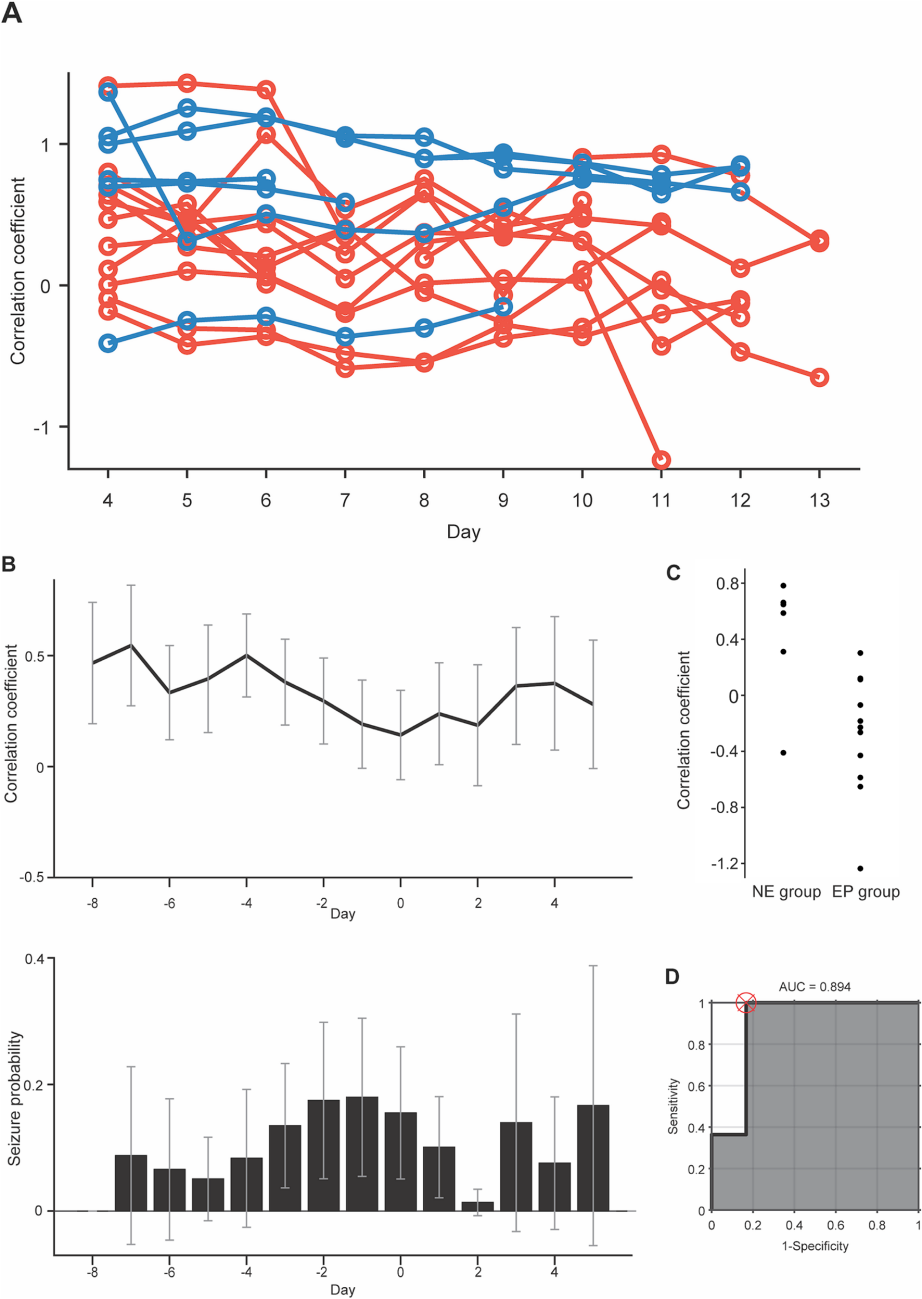
Corr‐RD values of long‐term recordings differ between the EP and NE groups. (A) Fisher z‐transformed correlation coefficients between the hippocampal ripple event rate and the cortical delta power are plotted for each day after electrode implantation for all patients in the EP (red) and NE (blue) groups. (B) Mean correlation coefficients aligned on the day with the minimum correlation coefficient are plotted with the mean frequency of the seizure events. The error bars represent the 95% confidence intervals. (C) The minimum value of the correlation coefficient between the hippocampal ripple event rate and the cortical delta power for each patient in each group is plotted. (D) ROC curve for classifying the NE and EP groups on the basis of the minimum value of the correlation coefficient.

The Corr‐RD values at night were compared between the two groups. The ripple event rate between 22:00 (lights out time) and 7:00 (lights on time) was analyzed. Over the 10 days of recordings, the Corr‐RD value during the night significantly differed between the EP (mean (SD): 0.202 (0.049)) and NE (0.665 (0.070)) groups (Figure 4A).

Among these patients, the seizure frequency increased as the Corr‐RD value decreased. When we averaged the Corr‐RD values of all patients by aligning the day with the minimum Corr‐RD value to day 0, the seizure frequency tended to increase 1–2 days before the day with the minimum Corr‐RD value (Figure 4B, Figures S4 and S7). Notably, the average seizure frequency and average Corr‐RD value did not change similarly among patients, and these values were not significantly correlated (*R* = 0.0423, *p* = 0.8763).

### 3.5 Detection of Hippocampal Sclerosis on the Basis of the Minimum Corr‐RD Value

We examined whether the two groups could be classified on the basis of the minimum Corr‐RD value following electrode implantation. The Corr‐RD values of the NE group were significantly higher than those of the EP group (NE group, 0.43 (0.44) (mean (SD)); EP group, −0.28 (0.43); *p* = 0.0057, *t*_15_ = −3.2, *n* = 17 hippocampi; *t* test). The results revealed that the two groups could be classified with 94.1% accuracy (AUC: 0.894; sensitivity: 100.0%; specificity: 83.3%) when a Corr‐RD value of 0.3 was used as the classification criterion, as depicted in Figure 4C,D (Figure S8; see Figure S9 for the number of ripples in both groups).

## 4 Discussion

The Corr‐RD value indicates epileptogenicity in the hippocampus. The Corr‐RDs at night were significantly lower in the EP group than in the NE group, which had hippocampal sclerosis. In this cohort, a Corr‐RD value of less than 0.3 could be used to predict hippocampal sclerosis with 94.1% accuracy. Despite the similar waveforms and frequency characteristics between the individuals in the NE and EP groups, nocturnal synchronization between the hippocampal ripples and cortical delta power is proposed as a biomarker of epileptogenicity in the hippocampus.

Previous studies have demonstrated that epileptic ripples are biomarkers of epileptogenicity in patients with epilepsy [5, 6, 7, 12, 26]. A greater percentage of ripples are found in the seizure onset zone than in normal tissue [4]; thus, the EP hippocampus can be inferred based on the ripple rate in unilateral mesial temporal lobe epilepsy [13]. However, in prior studies, pathological and physiological ripples were not distinguished. In this study, we hypothesized that epileptogenicity could be assessed by estimating Corr‐RD because normal hippocampal SWRs are strongly correlated with the depth of sleep, as indicated by delta power. In particular, we assessed the relationship between epileptogenicity and Corr‐RD by comparing two patient groups, those with and without hippocampal sclerosis, and increased epileptogenicity in each patient by reducing the use of antiepileptic agents. The Corr‐RD value tended to decrease with increasing seizure severity in response to reduced antiepileptic agent administration (Figure S7). Although the variation in the Corr‐RD values during treatment with reduced antiepileptic agents between the two groups was not statistically significant, the seizure frequency increased 1–2 days before the day with the minimum Corr‐RD value, which was determined on the basis of the hippocampal ripples and the cortical delta power. Thus, a decreased Corr‐RD value was suggested to indicate increased epileptogenicity. Notably, the maximum seizure frequency did not occur on the day with the minimum Corr‐RD value, implying that increased seizures did not directly cause the decreased correlation through epilepsy‐related EEG changes, such as an increase in interictal epileptic discharges (Figure S10). The results suggest that the increase in epileptogenicity owing to the reduction in antiepileptic agents resulted in an increase in the number of pathological ripples, thereby reducing the Corr‐RD value (Figure S11).

Interestingly, the Corr‐RD values were significantly different between patients with and without hippocampal sclerosis, suggesting that epileptogenicity can be identified by a decreased Corr‐RD value. For some patients in the EP group, the hippocampal ripple rate and cortical delta power were inversely correlated with the negative Corr‐RD value, suggesting that pathological ripples are not only out of phase with the delta power but also show the reversed phase. Theoretically, if pathological ripples are desynchronized with cortical delta power, the Corr‐RD value, which represents the combination of both pathological and physiological ripples, should approach zero rather than become negative as the number of pathological ripples increases. This reversed‐phase coupling between hippocampal ripples and cortical delta power may be related to hippocampal sclerosis. Moreover, an abnormal Corr‐RD value can be used to predict epileptogenicity rather than pathology and could thus be applicable to other conditions associated with hippocampal epilepsy, such as brain tumors and focal cortical dysplasia. As shown in Figure 3, the Corr‐RD value decreased around the days with epileptic seizures (also see Figure S4). These results suggest that a negative Corr‐RD value represents increased epileptogenicity. Further studies on various EP lesions in the hippocampus could be performed to determine the generalizability of these findings.

Although this study demonstrated a diurnal rhythm in ripple fluctuations, previous studies have reported diurnal variations in EP activity with 24‐h rhythms or even multiday rhythms [27, 28, 29]. Notably, increased interictal spikes have been reported during slow‐wave sleep [30, 31]. Additionally, interictal activity during wakefulness and rapid eye movement (REM) sleep has been used to predict epileptogenicity [30]. Similarly, increased interictal epileptiform discharges during nonrapid eye movement (NREM) sleep have been shown to be associated with increased delta power, affecting cognitive function [32, 33]. Moreover, other reports have highlighted an association between epilepsy and REM sleep, which differs from the relationship between epilepsy and NREM sleep [34, 35, 36]. Our results revealed two types of abnormal correlations within the EP group: hippocampal ripples were synchronized with cortical delta power with a decreasing correlation or with a reversed phase. These differences may explain the relationship between epileptogenicity and different sleep features, such as those observed in REM and NREM sleep. Large‐scale studies focusing on the synchrony between epileptic and normal EEG patterns and their relationships with various types of epilepsy are warranted.

In this study, we focused on the correlation between the diurnal fluctuations in ripple rates and delta power, revealing an abnormal correlation related to epileptogenicity. In addition to these circadian fluctuations, previous studies have revealed other relationships between ripples and cortical activity at different temporal and spatial scales. For example, during SWRs, hippocampal ripples show phase‐locking behavior with slow waves (0.5 to 4 Hz) in cortical and subcortical regions, and the strength of this phase–amplitude coupling varies across brain regions [15, 37]. Regarding cross‐frequency coupling, hippocampal ripples strongly couple with ripple‐band activity in the posterior parietal cortex and medial prefrontal cortex [38]. Although the present study focused on diurnal fluctuations on the basis of the hypothesis that the normal relationship between hippocampal ripples and delta power is altered by epileptogenicity, further studies on the temporal relationship between hippocampal ripples and cortical activities should be performed to identify their relationship with epileptogenicity and thus provide more precise biomarkers of epileptogenicity.

This study has certain limitations. First, bias may have been introduced owing to the retrospective nature of our study, despite our focus on patients with and without pathologically identified hippocampal sclerosis, necessitating validation with prospective studies. Second, the sample size was relatively small, limiting the generalizability of the findings. However, the reduced Corr‐RD value in the EP hippocampus demonstrates the importance of conducting a larger cohort study to confirm the generalizability of our findings. Third, although we could not investigate ripple activity on the basis of sleep staging due to the absence of electrooculogram and electromyogram data, which are essential for identifying specific sleep stages, we used cortical delta power as a proxy to reflect the circadian rhythm in the sleep–wake cycle. Fourth, hippocampal ripples and parahippocampal ripples were analyzed together. This analysis potentially includes variability due to differences in their physiological and pathological characteristics. Finally, we relied on invasive data acquisition techniques, which may not be feasible to use in all clinical settings. Distinguishing between pathological and physiological ripples is difficult, even with invasive techniques. Our results suggest that Corr‐RD during long‐term recordings is a useful biomarker for identifying epileptogenicity in the hippocampus without classifying each hippocampal ripple.

In conclusion, in this study, a novel biomarker for assessing the epileptogenicity of the hippocampus is provided using the correlation between hippocampal ripples and cortical delta power. These findings may lead to the development of improved diagnostic and treatment strategies for patients with refractory epilepsy.

## Author Contributions

**Takamitsu Iwata:** conceptualization, data curation, formal analysis, methodology, visualization, writing – original draft preparation, writing – review, and editing. **Takufumi Yanagisawa:** conceptualization, data curation, formal analysis, methodology, and writing – original draft preparation. **Yuji Ikegaya:** writing, reviewing, and editing. **Ryohei Fukuma:** methodology. **Satoru Oshino:** data curation, writing, reviewing, and editing. **Naoki Tani:** data curation, writing, reviewing, and editing. **Hui Ming Khoo:** data curation, writing, reviewing, and editing. **Hidenori Sugano:** writing, reviewing, and editing. **Yasushi Iimura:** writing, reviewing, and editing. **Hiroharu Suzuki:** writing, reviewing, and editing. **Haruhiko Kishima:** supervision, data curation, writing, reviewing, and editing.

## Consent

The authors confirm that written informed consent was obtained from the involved patients or, if appropriate, from the parent, guardian, or agent with the power of attorney regarding the involved patients and that they were given approval for this information to be published.

## Conflicts of Interest

The authors declare no conflicts of interest.

## Supporting information


Data S1.

## Data Availability

The datasets generated and analyzed during the current study are available on Figshare at https://doi.org/10.6084/m9.figshare.28375673.
