# Supplementary material for: Abnormal Synchronization Between Cortical Delta Power and Ripples in Hippocampal Sclerosis
Source: Ann Clin Transl Neurol. 2025 Mar 20;12(5):986–97. doi: 10.1002/acn3.70032 (PMC12093325; doi:10.1002/acn3.70032)
Supplement: Supplementary file 1 — Data S1. [file ACN3-12-986-s001.pdf]

Supplementary Materials for
‘Abnormal synchronization between cortical delta power and ripples in hippocampal sclerosis’

Takamitsu Iwata,[1] Takufumi Yanagisawa,[1,2] Yuji Ikegaya,[3,4,5] Ryohei Fukuma,[1] Satoru Oshino,[1] Naoki Tani,[1] Hui M. Khoo,[1] Hidenori Sugano,[6] Yasushi Iimura, [6] Hiroharu Suzuki, [6] and Haruhiko Kishima[1]

[1]Department of Neurosurgery, Graduate School of Medicine, Osaka University, Suita, Osaka, 565-0871, Japan
[2]Institute for Advanced Co-Creation Studies, Osaka University, Suita, Osaka, 565-0871, Japan
[3]Laboratory of Chemical Pharmacology, Graduate School of Pharmaceutical Sciences, The University of Tokyo, Tokyo 113-0033, Japan
[4]Institute for AI and Beyond, The University of Tokyo, Tokyo 113-0033, Japan
[5]National Institute of Information and Communications Technology, Center for Information and Neural Networks, Suita, Osaka, 565-0871, Japan

This PDF file includes the following:

Supplementary Figs. 1 to 11
Supplementary Table 1 and 2

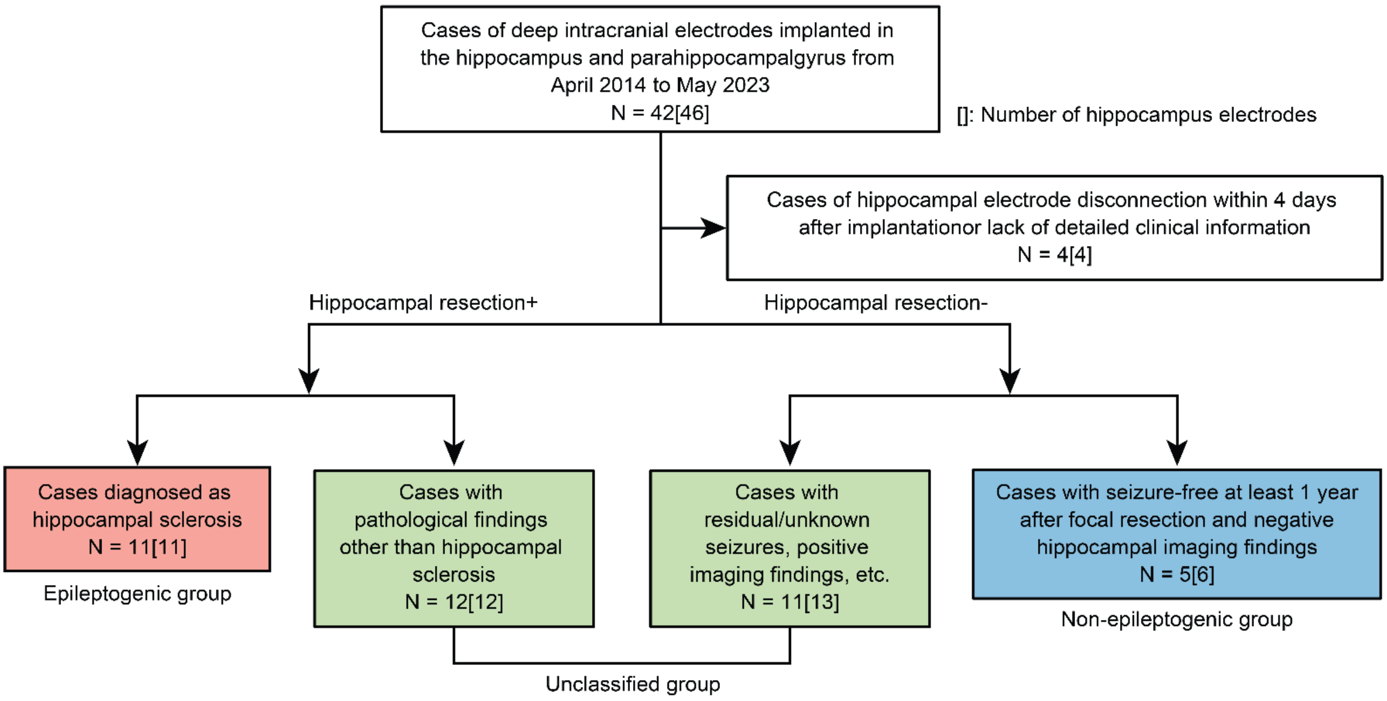

Supplementary Fig. 1 CONSORT diagram. We recorded the iEEG data of 42 patients from 2014 to 2023. The patients were classified into three groups: epileptogenic (EP) non-epileptogenic (NE), and unclassified (UC).

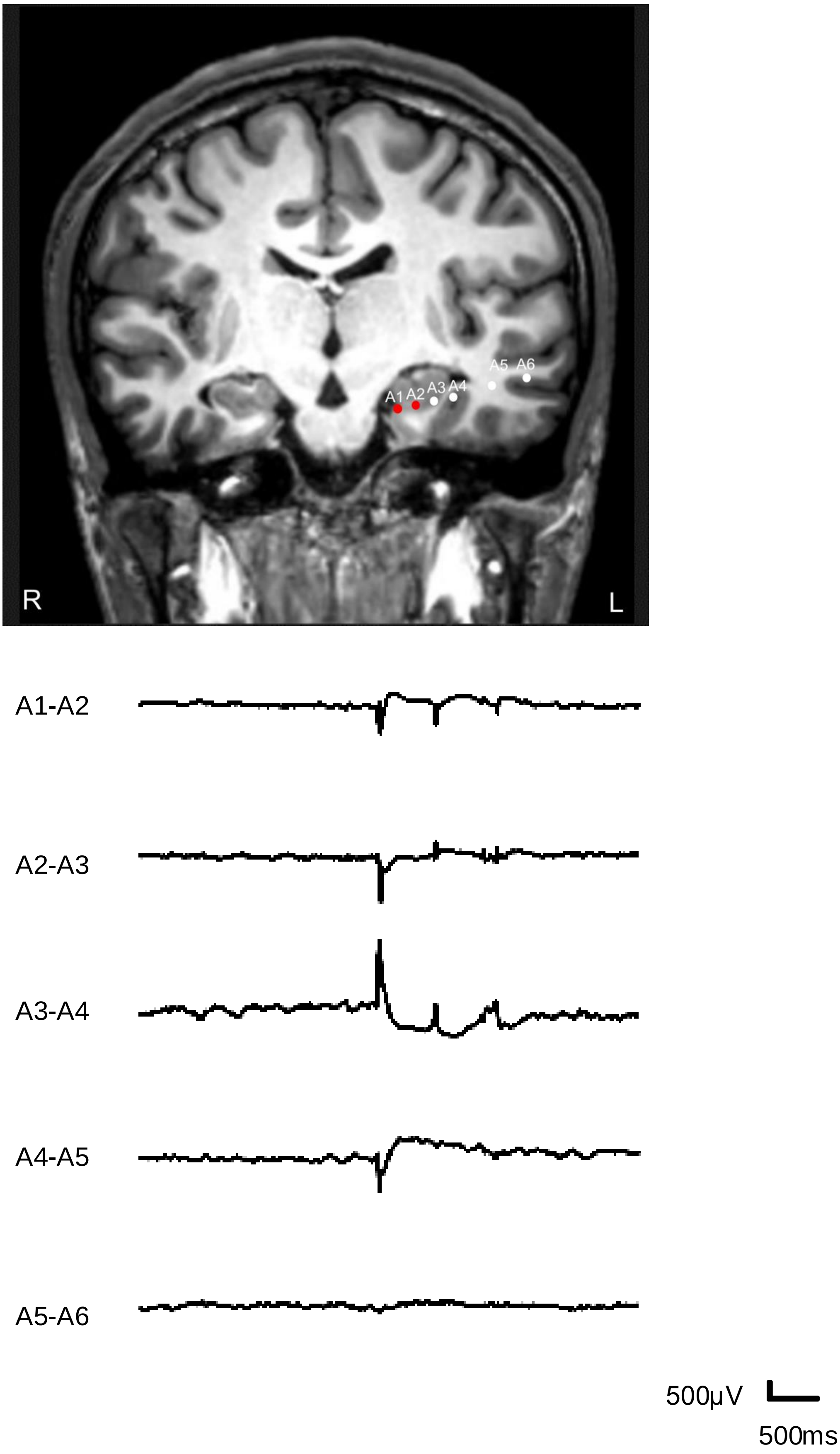

Supplementary Fig. 2 Bipolar potentials across all recorded channel.
This figure displays the waveforms recorded from all bipolar pairs in the electrode, providing a comprehensive view of the field potentials. Each trace represents the local field potential (LFP) recorded using a bipolar montage between adjacent contacts.

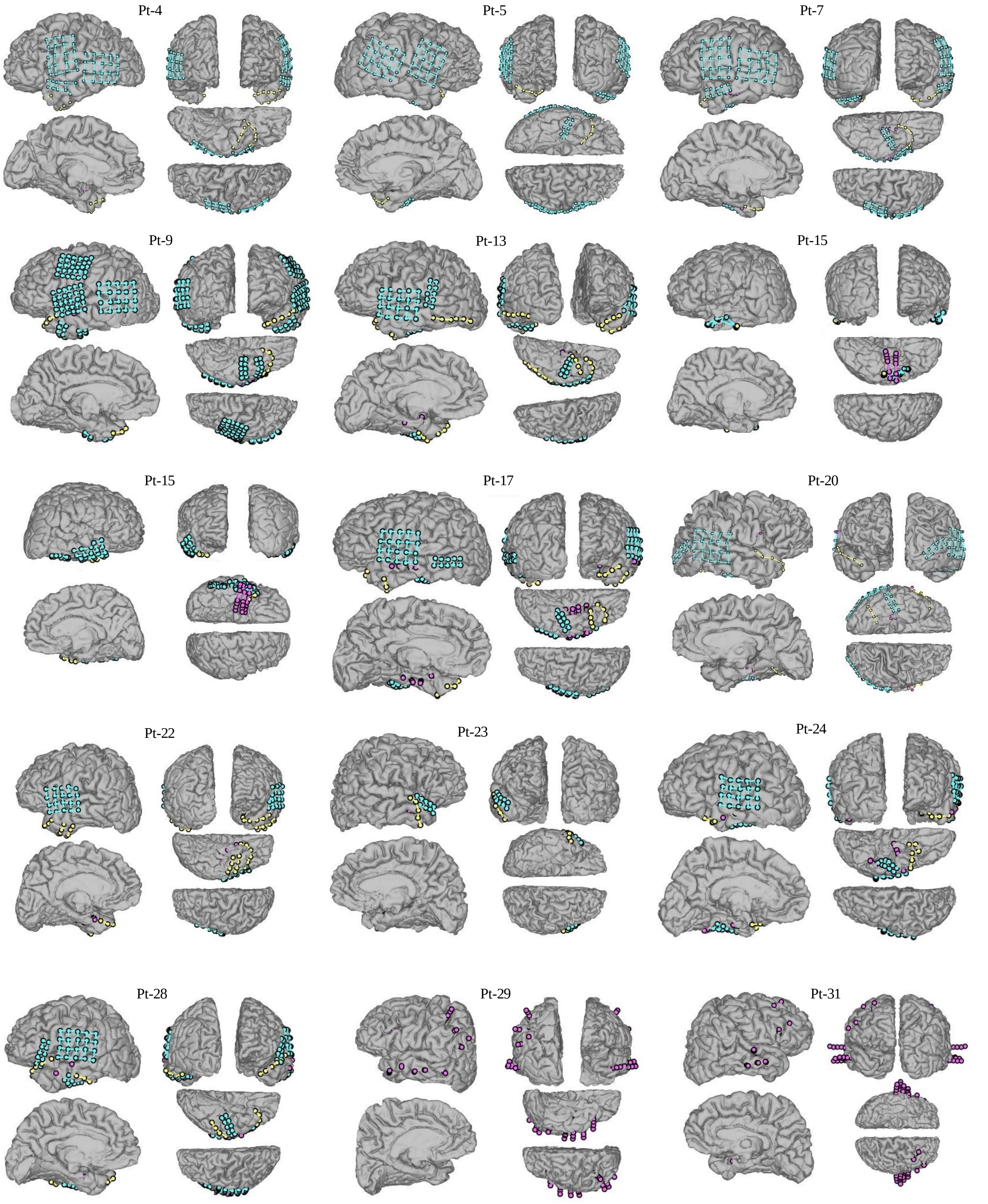

Supplementary Fig. 3 Intracranial electrode maps for all patients.

The light blue electrodes represent sheet-type cortical electrodes, the yellow electrodes represent single-row cortical electrodes, and the pink electrodes represent implanted depth electrodes.

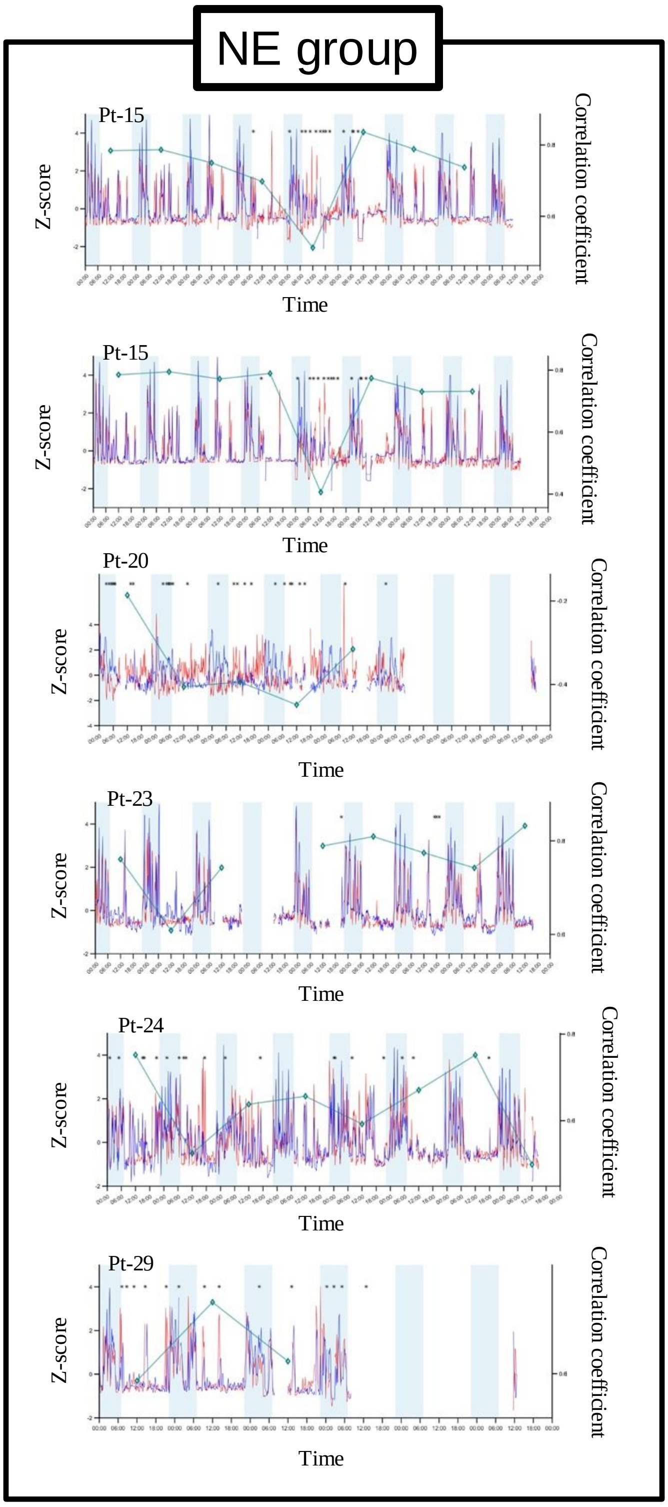

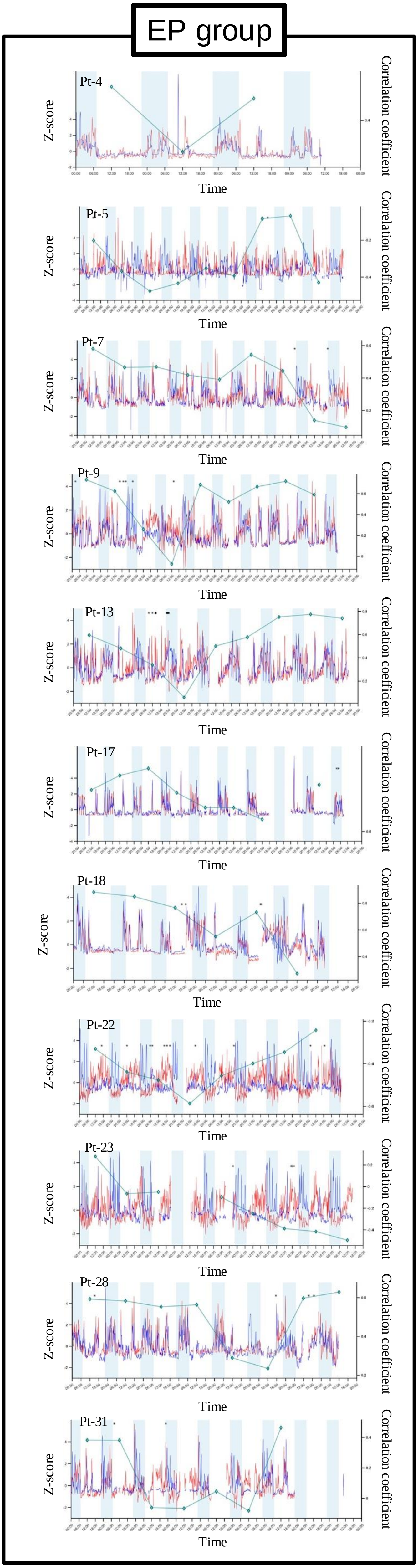

Supplementary Fig. 4 Ripple event rates, delta power and seizures for all patients during iEEG recordings after day 4. The time courses of the ripple event rates (red) and cortical delta power (blue) for all participants during the recording period after day 4. The light blue background indicates the time of day when the room was dark. Each square symbol represents the correlation coefficient between the ripple event rate and the delta power over the whole day. Each asterisk indicates the time of a seizure. The x-axis shows the time of day after intracranial electrode implantation, the left y-axis shows the Z score of the ripple event rate or delta band power, and the right y-axis shows the correlation coefficient between the two.

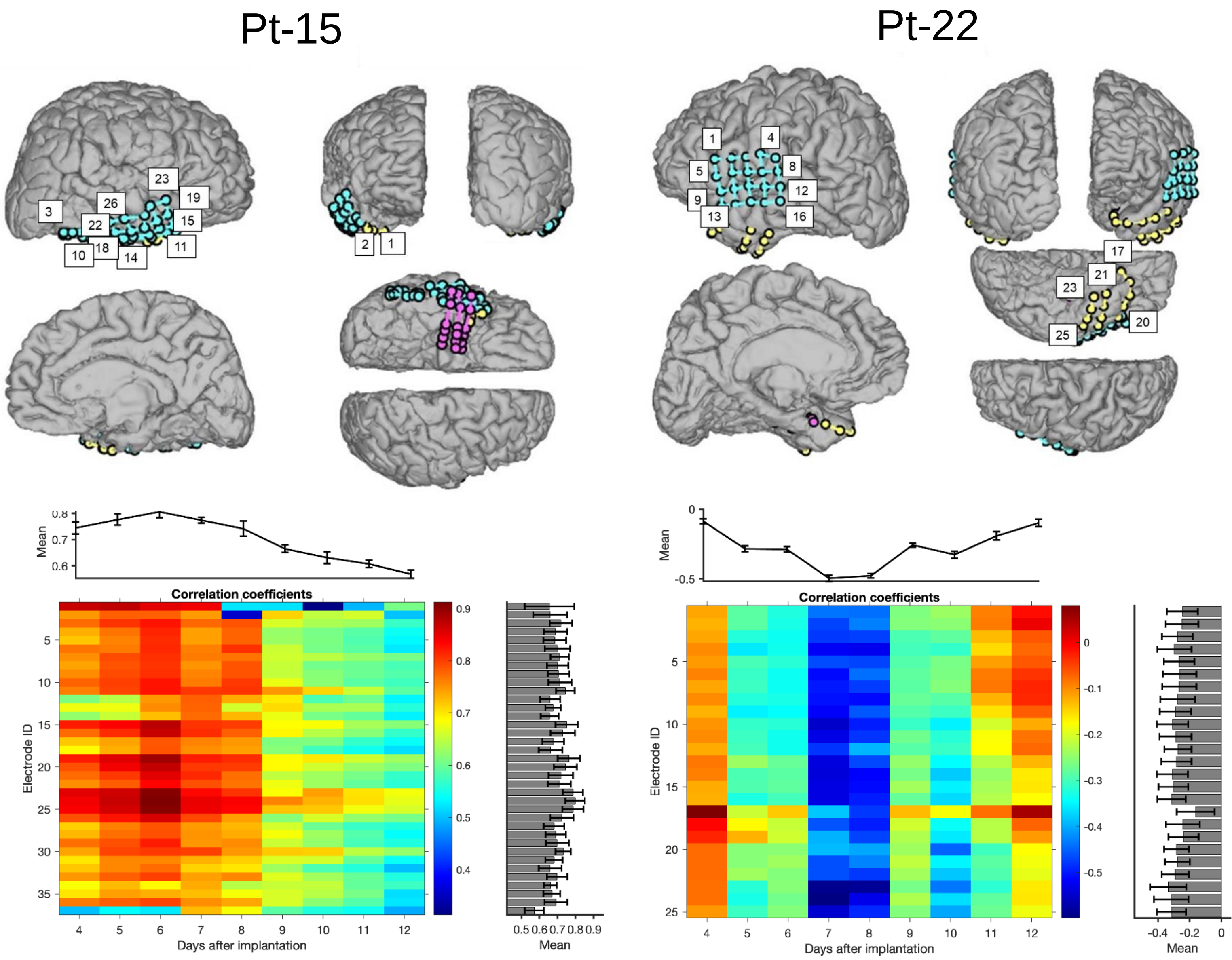

Supplementary Fig. 5 Variability in Corr-RD across channels and days.

We analyzed the Corr-RD values across different channels in representative cases from both the EP and NE groups. Our findings revealed channel-to-channel differences and day-to-day differences in Corr-RD (Pt-15; day-to-day; $F_{8,288}$ = 126.7, P = 1.1 * 10^{-89}, channel-to-channel; $F_{36,288}$ = 8.28, P = 1.7 * 10^{-27}, n =332; two-way ANOVA, Pt-22; day-to-day; $F_{8,192}$ = 232.26, P = 2.2 * 10^{-94}, channel-to-channel; $F_{24,192}$ = 4.87, P = 1.3 * 10^{-10}, n =224; two-way ANOVA).

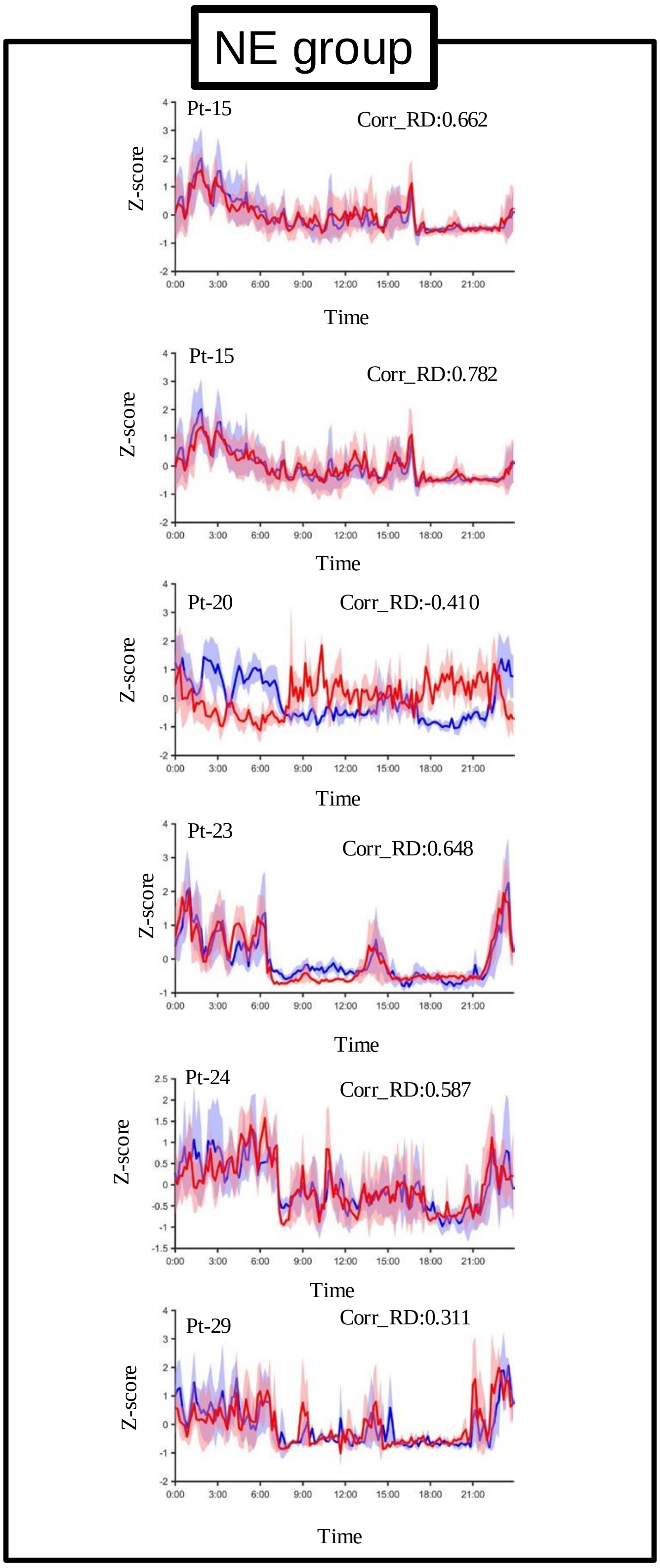

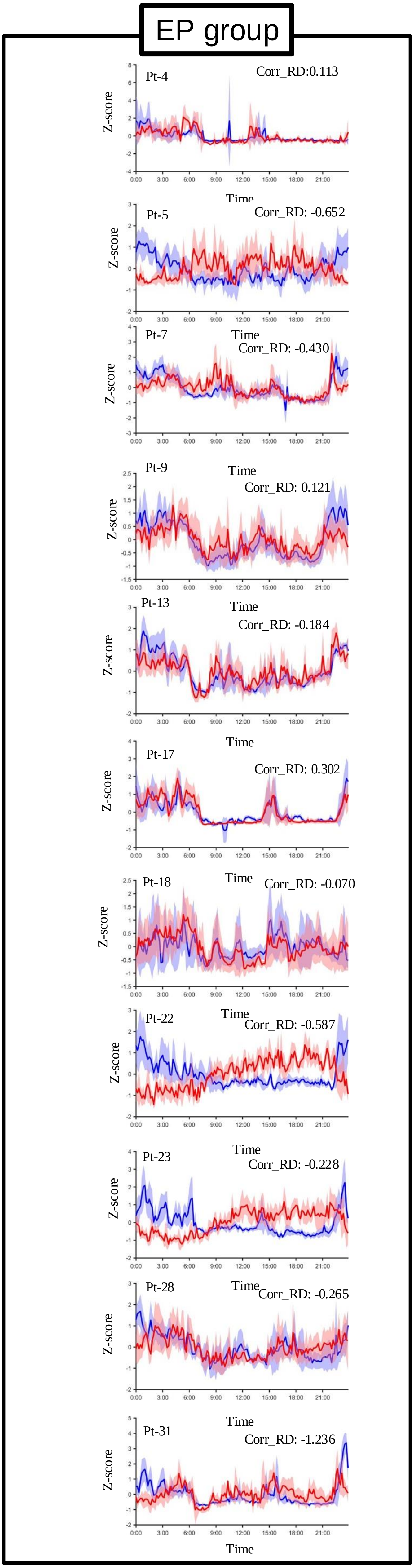

Supplementary Fig. 6 Mean ripple event rates and delta power of each patient based on the iEEG recordings after day 4. The lines show the means and 95% confidence intervals of the Z score ripple event rates (red) and delta powers (blue) over 24 hr for all patients in the NE group and EP group. The x-axis represents time, and the y-axis represents the z score of the ripple event rate or delta band power.

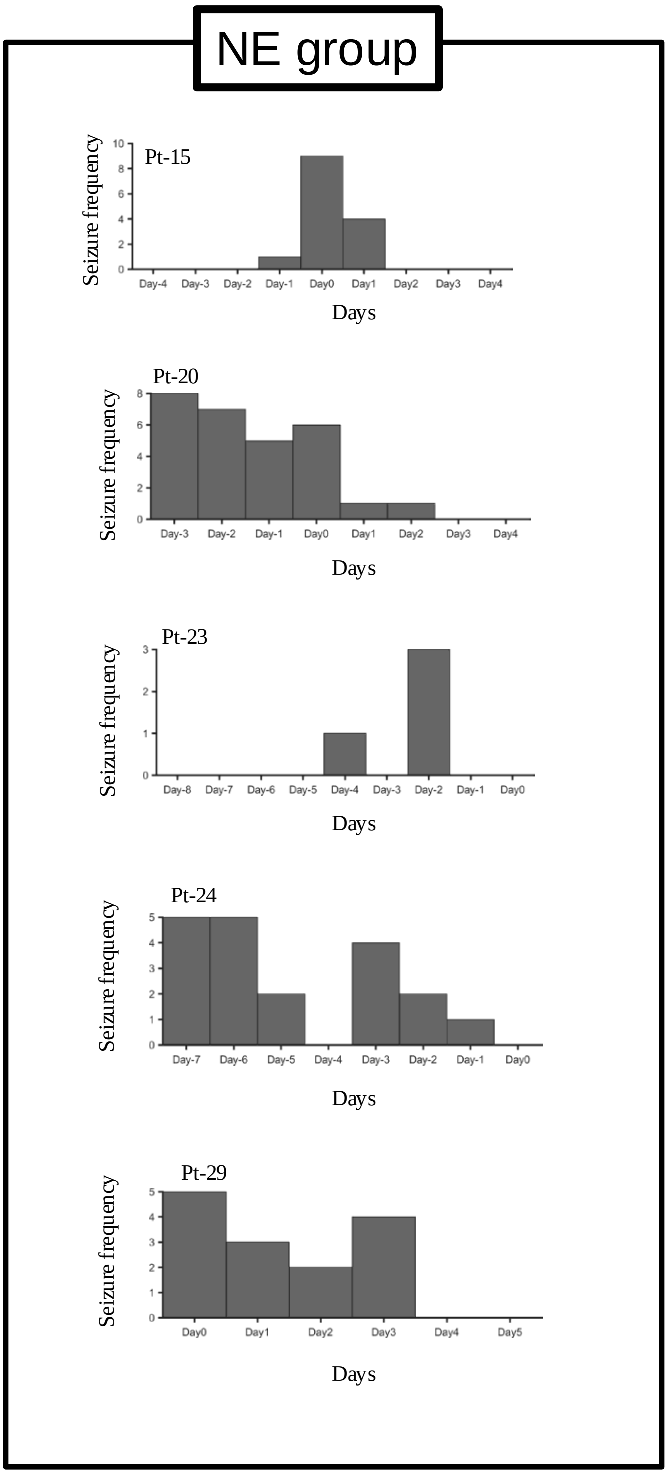

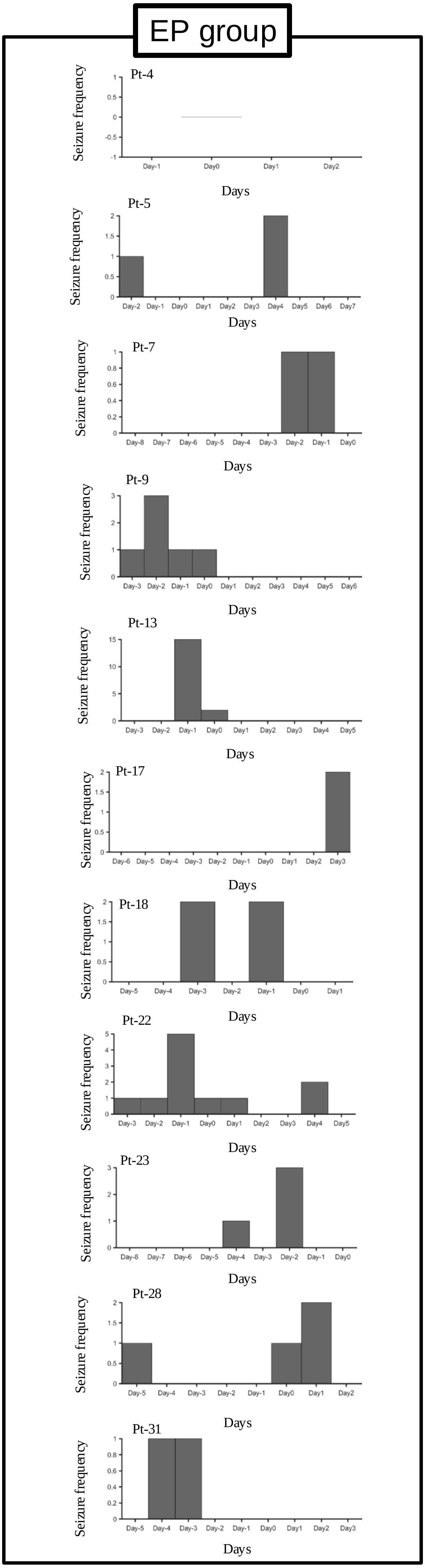

Supplementary Fig. 7 Seizure event frequency around the day with the minimum correlation coefficient. The seizure event frequency aligned with the day on which the minimum correlation coefficient was obtained was plotted for all patients in the NE group and EP group. The bar for each day shows the frequency of epileptic seizures for that day. The x-axis shows the days elapsed from the date on which the minimum correlation coefficient was obtained, and the y-axis shows the frequency of epileptic seizures.

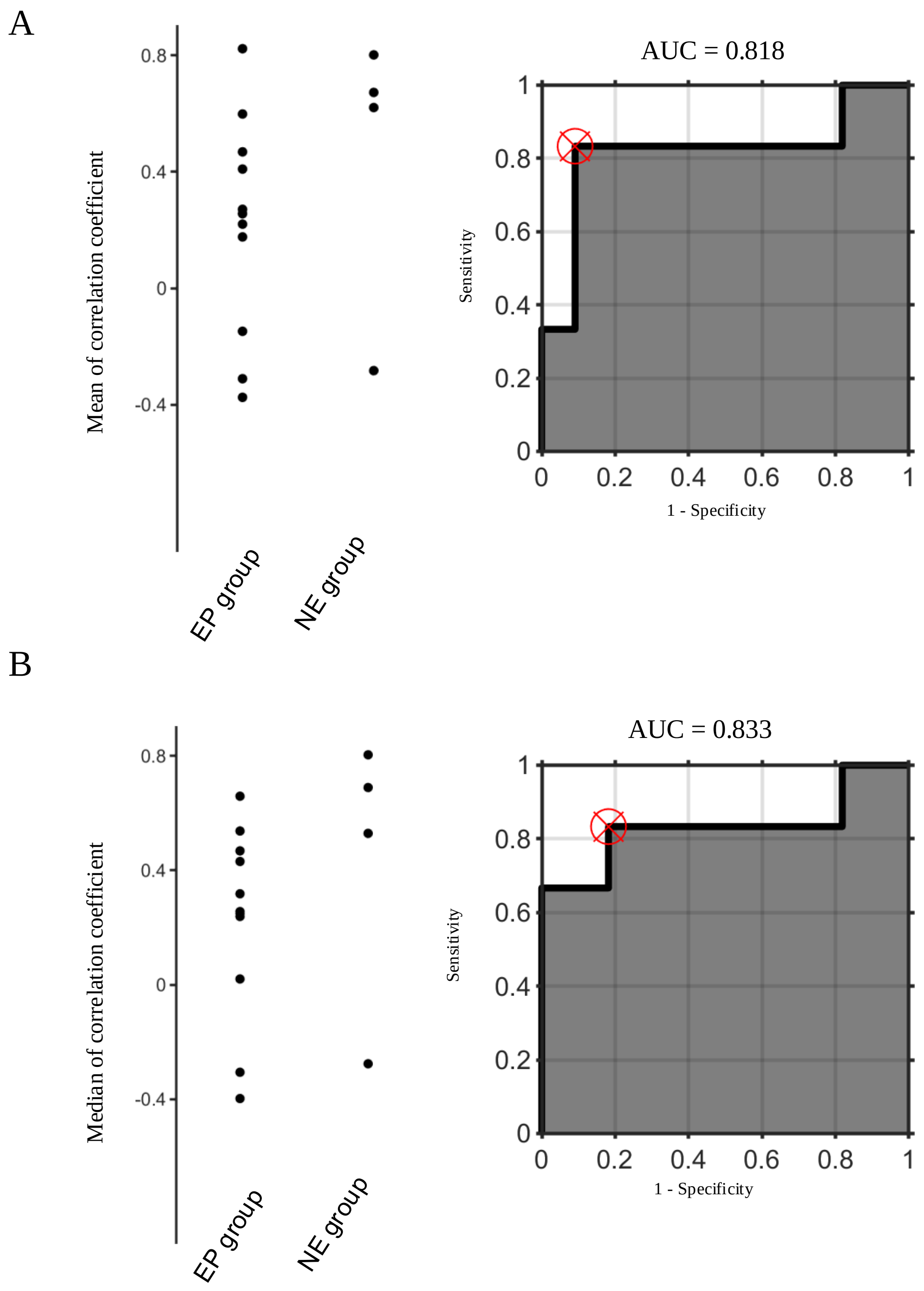

Supplementary Fig. 8 Mean and median value of the correlation coefficient and ROC to classify two groups based on the values. (**A**) The mean value of the correlation coefficient between the ripple event rates and the delta powers for each patient was plotted for each group (left panel). ROC curve (AUC=0.818) to classify the NE group and EP group based on the mean value of the correlation coefficient (right panel). (**B**) The median value of the correlation coefficient between the ripple event rates and the delta powers for each patient was plotted for each group (left panel). ROC curve (AUC=0.833) to classify the NE group and EP group based on the median value of the correlation coefficient (right panel).

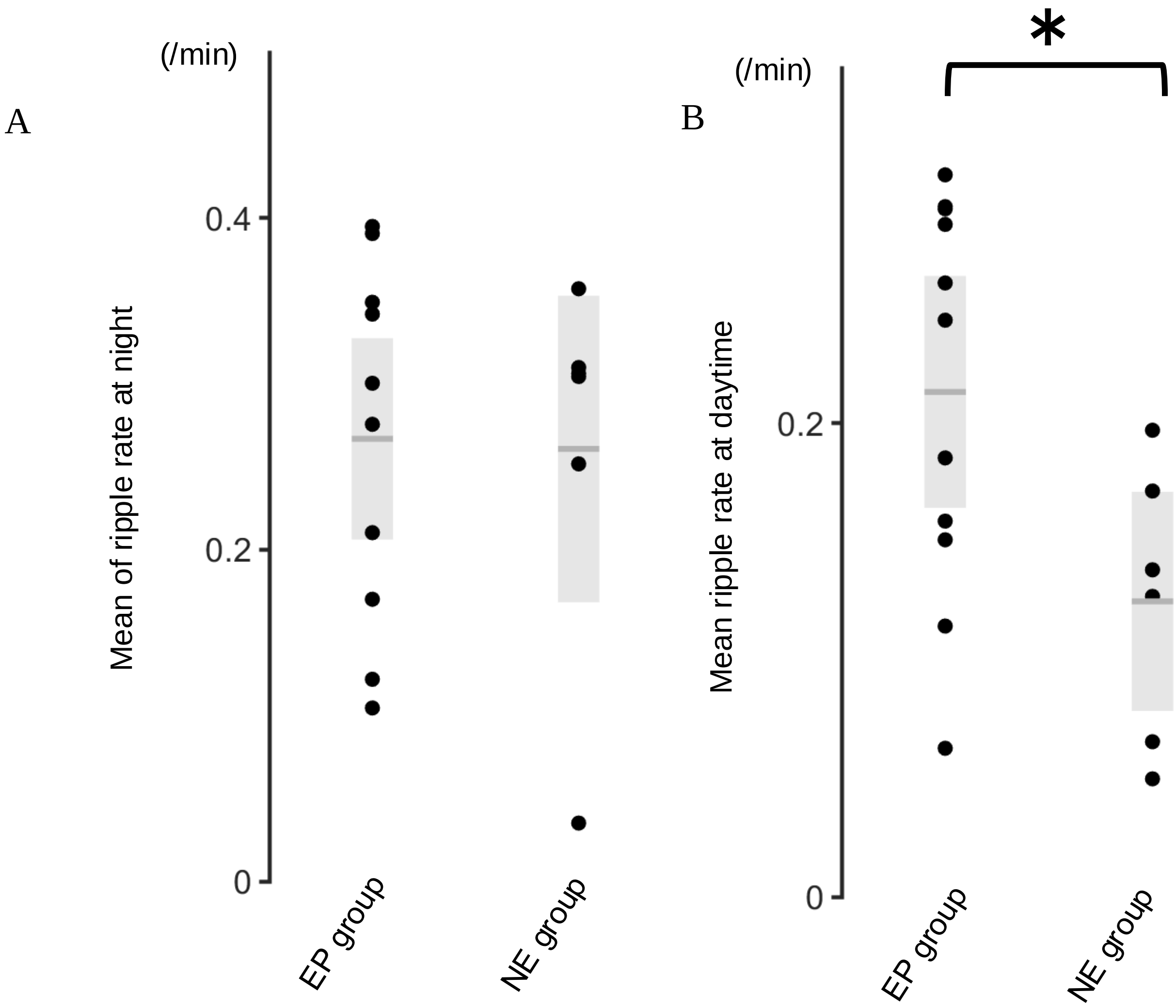

Supplementary Fig. 9 Comparison of ripple rates between the two groups during the daytime and nighttime periods.

We compared ripple rates between the two groups during the daytime and nighttime periods separately. The results revealed that while ripple rates during the daytime were greater in the EP group, there was no significant difference in ripple rates between the EP and NE groups during the nighttime (daytime NE group, 0.12 (0.06) (mean (SD)); daytime EP group, 0.21 (0.08); P = 0.036, t_{15} = -2.3, n =17 hippocampi; t test, night NE group, 0.26 (0.12); night EP group, 0.27 (0.10); P = 0.91, t_{15} =-0.11, n =17 hippocampi; t test).

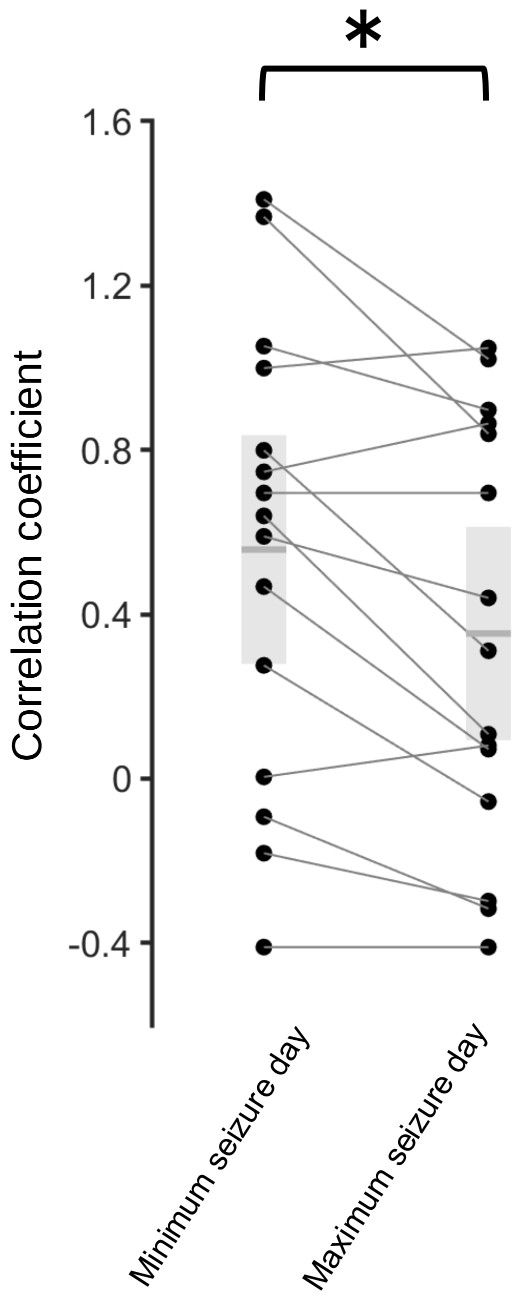

Supplementary Fig. 10 Comparison of the mean correlation coefficients on the days with the maximum and minimum number of seizures.

We compared the mean correlation coefficients on the days with the maximum and minimum number of seizures during the intracranial electrode monitoring period . The first day of analysis was defined as the day with the minimum number of seizures.

There were no significant differences between the values (minimum number of seizures, 0.35 (0.51) (mean (SD)); maximum number of seizures, 0.56 (0.55); $P = 0.0036$, $t_{14} = -3.5$, n =15 hippocampi; paired t test).

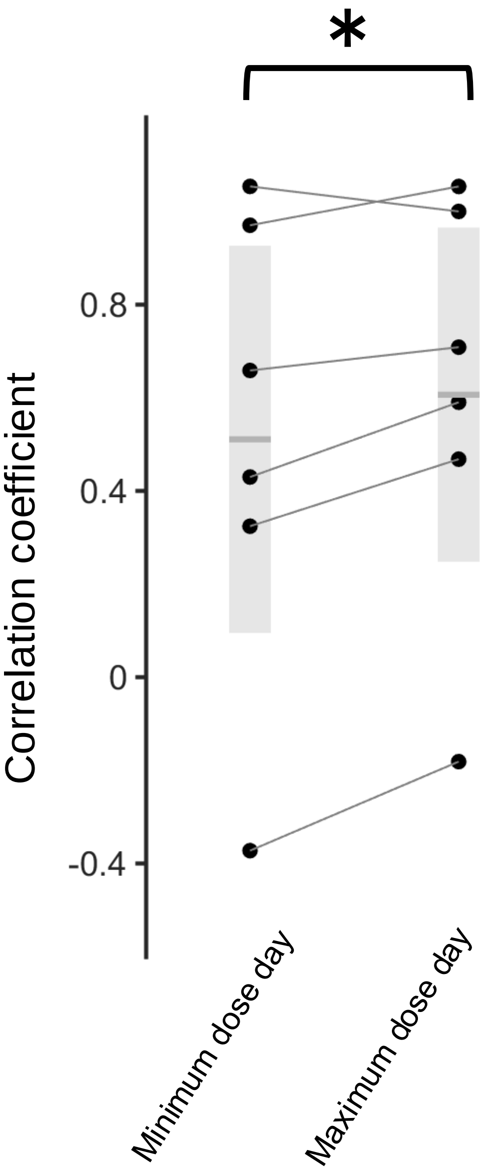

Supplementary Fig. 11 Comparison of Corr-RD values on the days with the maximum and minimum antiseizure medication doses.

We conducted an analysis comparing the mean correlation coefficient values on the days with the maximum and minimum antiseizure medication doses for participants for whom these days were clearly identifiable. The Corr-RD values on the days with the maximum antiseizure medication doses were greater than those on the days with the minimum doses (minimum dose day, 0.51 (0.52) (mean (SD)); maximum dose day, 0.61 (0.45); P = 0.047, t_5 = 2.6, n =6 hippocampi; paired t test).

Supplementary table 1 Patient characteristics

	Value
Age (years)	28.3 ± 12.5
Sex	F 14 M 24
Side of electrode implantation	right 11 left 23 bilateral 4
Region of implantation	parahippocampus 17 hippocampus 25
Electrode implantation period (days)	10.8 ± 2.0
Type of electrodes	ECoG 29 SEEG 9
Age at initial seizure (years)	14.2 ± 10.3
Disease duration (years)	14.1 ± 9.8

Supplementary table 2 Information on the amount of anti-epileptic drug for each patient

Pt ID	Anti-epilepti c drug	pre ope	Day 1	Day 2	Day 3	Day 4	Day 5	Day 6	Day 7	Day 8	Day 9	Day 10	Day 11	Day 12	Day 13	Day 14	Day 15	Day 16
1	VPA	1000	1000	→	→	0	→	→	→	→	→	→	→	→	→	→	600	1000
	LEV	2000	2000	500	0	→	→	1000	→	500	0	1000	→	3000	→	→	→	→
2	LEV	2500	2500	→	→	→	→	→	→	→	→	→	→	→	→	→		
3	CBZ	400	400	→	→	→	300	100	0	→	→	→	→	→				
	CLB	30	30	→	→	→	→	→	→	→	0	→	→	→				
4	CBZ	200	200	→	→	100	0	→	100	200	200	→	→	→				
	LEV	1000	1000	750	500	→	→	250	→	1000	1000	→	→	→				
5	GBP	2000	2000	→	→	→	→	→	1600	200	0	→	800	2000				
	TPM	75	75	→	→	→	→	→	→	→	→	→	→	→				
	LEV	3000	1000	1500	2000	→	→	→	→	→	→	→	→	→				
6	PHT	225	225	100	0	→	→	→	→	→	→	→	200	→	→			
	CLB	5	5	→	→	0	→	→	→	→	→	→	5	→	→			
	TPM	300	100	0	→	→	→	→	→	→	→	→	300	→	→			
7	CLB	20	20	→	10	→	→	→	→	→	→	→	→	20	→			
	LTG	75	0	→	→	→	→	→	→	→	→	→	→	75	→			
8	CLB	15	15	→	→	→	→	→	→	→	→	→	→	→				
	LTG	350	350	→	→	→	→	→	→	→	→	→	→	→				
	LEV	1500	0	→	→	→	→	→	→	→	→	750	1500	→				
	LCM	400	400	→	→	→	→	→	→	200	0	200	400	→				
9	CZP	1	1	→	→	→	→	→	→	→	→	→	→	→				
	VPA	1200	1200	→	→	→	→	→	→	→	→	→	→	→				
	GBP	600	0	→	→	→	→	→	→	→	→	→	→	→				
	LEV	2000	1000	→	→	→	500	0	→	→	→	1000	2000	→				
	LCM	100	0	→	→	→	→	→	→	→	→	→	→	→				
10	PB	60	60	60	30	15	0	→	→	30	60							
11	NZP	5	5	→	→	→	→	→	→	→	→	→	→	→	→	→	→	
	CBZ	400	400	→	→	→	→	→	→	→	→	→	→	→	→	→	→	
	LCM	400	400	300	100	0	→	→	→	200	→	400	→	→	→	→	→	
12	PHT	100	100	→	→	→	→	50	0	→	→	→	50	100	→	→		
	ST	300	300	→	→	→	→	→	→	→	→	→	→	→	→	→		
	CBZ	600	600	500	400	→	→	200	0	→	→	→	→	→	→	600		
	LTG	500	500	→	→	→	→	→	→	→	475	450	475	500	→			
13	CBZ	900	600	→	→	400	200	→	900	→	→	→	→	→	→			
	VPA	400	400	→	→	→	→	→	→	→	→	→	→	→	→			
	ZNS	50	50	→	→	→	→	→	→	→	→	→	→	→	→			
	GBP	400	400	→	→	→	→	→	→	→	→	→	→	→	→			
14	VPA	1000	1000	→	→	→	→	→	→	800	500	→	→	→	700			
	LTG	70	70	→	→	→	→	→	→	→	→	→	→	→	→			
	LCM	25	25	→	→	→	→	→	→	→	→	→	→	→	→			
15	CBZ	400	400	→	→	→	200	100	0	→	400	→	→	→	→			
	LTG	200	200	→	150	100	→	→	→	→	200	→	→	→	→			
	PER	2	2	→	→	→	→	→	→	→	→	→	→	→	→			
	LCM	200	200	→	→	→	→	→	→	→	→	→	→	→	→			
16	CBZ	400	400	→	→	200	0	→	→	→	→	→	→	→	400			
	LEV	2000	1000	0	→	→	→	→	→	→	→	→	→	→	2000			
	PER	2	2	→	→	→	→	→	4	2	→	→	→	→	→			
17	CBZ	400	400	→	→	→	0	→	→	→	→	→	→	→	400			
	LTG	200	200	→	→	→	→	0	→	→	→	→	→	→	100			
18	LTG	210	210	→	→	→	160	60	→	110	→	→	210	→				
	PER	6	6	→	→	→	→	→	→	→	→	→	→	→				
	LCM	400	400	300	200	100	50	0	→	200	400	→	→	→				
19	LEV	1000	500	1000	1000	750	500	750	1000	→	→	→	→	→	→			
	LTG	200	→	→	→	→	→	→	→	→	→	→	→	→	→			
	LCM	400	0	→	→	→	→	→	→	→	→	→	→	→	→			
	CLB	10	→	→	→	→	→	→	→	→	→	→	→	→	→			
20	LEV	1000	→	→	→	→	→	→	→	→	→	→	→					

	CBZ	450	→	→	→	→	→	→	→	→	→	→	→			
	VPA	700	→	→	→	→	→	→	→	→	→	→	→			
	ZNS	300	→	→	→	→	→	→	→	→	→	→	→			
	CLB	30	→	→	→	→	→	→	→	→	→	→	→			
21	LEV	2500	2000	→	→	→	1500	1000	→	→	→	2500	→	→	→	
	LCM	250	→	200	→	250	150	→	→	→	→	250	→	→	→	
	CLB	3	→	→	→	→	→	→	→	→	→	→	→	→	→	
22	LEV	1000	500	1000	→	→	→	→	→	→	→	→	→	→	→	→
	LCM	300	150	250	200	0	→	→	→	→	→	→	→	→	→	→
23	LEV	3000	1000	→	→	→	→	→	→	→	→	→	→	→	→	
	PER	8	→	→	→	→	→	→	→	→	→	→	→	→	→	
	LCM	200	→	→	→	→	→	→	→	→	→	→	→	→	→	
24	LEV	2000	1500	2000	→	→	→	→	→	→	→	→	→	→	→	
	PER	2	→	→	→	→	→	→	→	→	→	→	→	→	→	
	LCM	100	100	200	200	→	→	→	→	→	→	→	→	→	→	
25	LEV	2500	→	1500	500	→	→	→	→	750	1000	1500	1750	2000		
	PER	8	→	→	→	→	→	2	→	4	4	→	6	8		
	LTG	400	→	→	→	→	→	200	100	200	300	→	400	400		
26	LTG	200	200	→	→	100	→	→	50	→	75	125	200			
	LCM	400	0	→	→	→	→	→	→	→	200	400	→			
27	LEV	1000	750	250	0	750	500	250	→	750	500	250	→	→	0	500
	LTG	125	75	25	→	50	25	→	→	50	→	25	→	50	0	25
28	PB	90	90	→	→	→	→	→	→	→	→	→	→	→		
	VPA	800	800	→	→	→	→	→	→	→	→	→	→	→		
	LTG	200	200	→	→	→	→	→	→	→	→	→	→	→		
	LEV	1500	1500	→	→	→	→	→	→	→	→	→	→	→		
	LCM	400	400	→	→	→	→	→	→	→	→	→	→	→		
29	LCM	400	200	200	100	0	100	100	0	200	400					
	VPA	1000	600	400	400	200	200	300	100	400	800					
	CLB	10	5	5	0	0	0	5	0	0	20					
30	LEV	3000	1500	0	→	→	→	→	→	1500	3000	→	→	→	→	
	CBZ	500	500	→	→	→	→	→	→	→	→	→	→	→	→	
31	LCM	200														
	PHT	275														
	CLB	15														
32	LEV	2250	2250	1500	1000	→	1750	2250	2250	→	→	→	→			
	VPA	800	800	→	→	→	→	→	→	→	→	→	→			
	LCM	400	400	→	→	→	→	→	→	→	→	→	→			
	PER	4	4	→	→	→	→	→	→	→	→	→	→			
33	LEV	500	500	250	→	500	250	0	→	→	→	250	500	→		
	LCM	400	400	→	→	300	200	150	100	→	200	350	400	→		

LEV: Levetiracetam, PER: Perampanel, VPA: Valproic Acid, CBZ: Carbamazepine, CLB: Clobazam, GBP: Gabapentin, TPM: Topiramate, PHT: Phenytoin, LTG: Lamotrigine, CZP: Clonazepam, LCM: Lacosamide, PB: Phenobarbital, NZP: Nitrazepam, ST: Sulthiame, ZNS: Zonisamide. The numbers indicated in the antiepileptic drug column represent the daily dosage in milligrams (mg).
